# Supplementary material for: HIVprotI: an integrated web based platform for prediction and design of HIV proteins inhibitors
Source: J Cheminform. 2018 Mar 9;10:12. doi: 10.1186/s13321-018-0266-y (PMC5845081; doi:10.1186/s13321-018-0266-y)
Supplement: Supplementary file 2 — Additional file 2. Source code of HIVProtI web server. [file 13321_2018_266_MOESM2_ESM.zip › HIVprotI_Source-code/hivprotI/weka-3-6-6/doc/weka/core/Debug.SimpleLog.html]

Debug.SimpleLog


---


|  |  |  |
| --- | --- | --- |
| **Overview** \| **Package** \| **Class** \| **Tree** \| **Deprecated** \| **Index** \| **Help** \| **Weka's home** | |  |
| **PREV CLASS**   **NEXT CLASS** | **FRAMES**    **NO FRAMES**     **All Classes** |  |
| SUMMARY: NESTED \| FIELD \| CONSTR \| METHOD | DETAIL: FIELD \| CONSTR \| METHOD |  |


---


## weka.core Class Debug.SimpleLog

```
java.lang.Object
  weka.core.Debug.SimpleLog
```

**All Implemented Interfaces:**: java.io.Serializable, RevisionHandler

**Enclosing class:**: Debug

---

``` public static class Debug.SimpleLog extends java.lang.Object implements java.io.Serializable, RevisionHandler ```

A little, simple helper class for logging stuff. Uses simple file access
and not the java.util.logging stuff (see Log for that). Uses the
writeToFile methods of the Debug class.

**See Also:**: `Debug.Log`, `Debug.writeToFile(String, String)`, `Debug.writeToFile(String, String, boolean)`, Serialized Form

---

**Constructor Summary**

`Debug.SimpleLog()`
  
          default constructor, uses only stdout

`Debug.SimpleLog(java.lang.String filename)`
  
          Creates a logger that writes into the specified file.

`Debug.SimpleLog(java.lang.String filename,
boolean append)`
  
          Creates a logger that writes into the specified file.


| **Method Summary** | |
| --- | --- |
| `java.lang.String` | `getFilename()`             returns the filename of the log, can be null |
| `java.lang.String` | `getRevision()`             Returns the revision string. |
| `void` | `log(java.lang.String message)`             logs the given message to the file |
| `void` | `logSystemInfo()`             a convenience method for dumping the current system info in the log file |
| `java.lang.String` | `toString()`             returns a string representation of the logger |

**Methods inherited from class java.lang.Object**

`equals, getClass, hashCode, notify, notifyAll, wait, wait, wait`

**Constructor Detail**

### Debug.SimpleLog

```
public Debug.SimpleLog()
```

:   default constructor, uses only stdout

---


### Debug.SimpleLog

```
public Debug.SimpleLog(java.lang.String filename)
```

:   Creates a logger that writes into the specified file. Appends to the
    file by default.

    **Parameters:**: `filename` - the file to write to, if null then only stdout is used

---


### Debug.SimpleLog

```
public Debug.SimpleLog(java.lang.String filename,
                       boolean append)
```

:   Creates a logger that writes into the specified file. Appends to the
    file by default.

    **Parameters:**: `filename` - the file to write to, if null then only stdout is used: `append` - if false, the file will be deleted first


**Method Detail**

### getFilename

```
public java.lang.String getFilename()
```

:   returns the filename of the log, can be null

    :   **Returns:**: the filename of the log

---


### log

```
public void log(java.lang.String message)
```

:   logs the given message to the file

    :   **Parameters:**: `message` - the message to log

---


### logSystemInfo

```
public void logSystemInfo()
```

:   a convenience method for dumping the current system info in the
    log file

    :   **See Also:**: `SystemInfo`

---


### toString

```
public java.lang.String toString()
```

:   returns a string representation of the logger

    :   **Overrides:**: `toString` in class `java.lang.Object`
    :   **Returns:**: a string representation of the logger

---


### getRevision

```
public java.lang.String getRevision()
```

:   Returns the revision string.

    :   **Specified by:**: `getRevision` in interface `RevisionHandler`
    :   **Returns:**: the revision


---


|  |  |  |
| --- | --- | --- |
| **Overview** \| **Package** \| **Class** \| **Tree** \| **Deprecated** \| **Index** \| **Help** \| **Weka's home** | |  |
| **PREV CLASS**   **NEXT CLASS** | **FRAMES**    **NO FRAMES**     **All Classes** |  |
| SUMMARY: NESTED \| FIELD \| CONSTR \| METHOD | DETAIL: FIELD \| CONSTR \| METHOD |  |


---
